# PEEK Cages versus PMMA Spacers in Anterior Cervical Discectomy: Comparison of Fusion, Subsidence, Sagittal Alignment, and Clinical Outcome with a Minimum 1-Year Follow-Up

**DOI:** 10.1155/2014/398396

**Published:** 2014-07-02

**Authors:** Jan-Helge Klingler, Marie T. Krüger, Ronen Sircar, Evangelos Kogias, Christoph Scholz, Florian Volz, Christian Scheiwe, Ulrich Hubbe

**Affiliations:** Department of Neurosurgery, Freiburg University Medical Center, 79106 Freiburg, Germany

## Abstract

*Purpose*. To compare radiographic and clinical outcomes after anterior cervical discectomy in patients with cervical degenerative disc disease using PEEK cages or PMMA spacers with a minimum 1-year follow-up. *Methods*. Anterior cervical discectomy was performed in 107 patients in one or two levels using empty PEEK cages (51 levels), Sulcem PMMA spacers (49 levels) or Palacos PMMA spacers (41 levels) between January, 2005 and February, 2009. Bony fusion, subsidence, and sagittal alignment were retrospectively assessed in CT scans and radiographs at follow-up. Clinical outcome was measured using the VAS, NDI, and SF-36. *Results*. Bony fusion was assessed in 65% (PEEK cage), 57% (Sulcem), and 46% (Palacos) after a mean follow-up of 2.5 years. Mean subsidence was 2.3–2.6 mm without significant differences between the groups. The most pronounced loss of lordosis was found in PEEK cages (−4.1°). VAS was 3.1 (PEEK cage), 3.6 (Sulcem), and 2.7 (Palacos) without significant differences. Functional outcome in the PEEK cage and Palacos group was superior to the Sulcem group. *Conclusions*. The substitute groups showed differing fusion rates. Clinical outcome, however, appears to be generally not correlated with fusion status or subsidence. We could not specify a superior disc substitute for anterior cervical discectomy. This trial is registered with DRKS00003591.

## 1. Introduction

Cervical degenerative disc disease includes disc herniation and spinal canal stenosis and is a common cause of neck pain with radicular and myelopathic symptoms. Surgical treatment is indicated if conservative treatment failed or neurological deficits occurred. Depending on the location and extent of the pathology, an anterior or posterior approach has to be considered. If cervical degenerative disc disease is limited to one or two levels, an anterior cervical discectomy (ACD) is usually performed including decompression of neural structures and implantation of a disc substitute. Traditionally, no disc substitute or an iliac crest autograft was used. Iliac crest autograft was found to provide higher fusion rates than other substitutes and also led to relevant donor site morbidity [1]. As part of further development, bone cement was implanted into the intervertebral disc space in order to restore segmental height and to avoid donor site morbidity [2–8]. Currently, spine surgeons are increasingly using intervertebral cages, which initially consisted of carbon [9–11] or titanium [2, 5, 8, 12–18] and later consisted mostly of polyetheretherketone (PEEK) [14, 16, 18–27]. The implantation of an artificial disc is a further surgical option; a clear superiority over ACD and fusion, however, has not been specified [28].

A direct comparative study between PEEK cages and the bone cement polymethylmethacrylate (PMMA) as cervical disc substitute has not been reported in the literature so far. The aim of this study was to compare radiographic and clinical outcomes after ACD in patients with cervical degenerative disc disease using PEEK cages or PMMA spacers with a minimum 1-year follow-up. Primary outcome measures were bony fusion and pain level at follow-up. Secondary outcome measures were degree of subsidence, loss of lordosis, functional outcome (questionnaires), and number of reoperations. We hypothesized that fusion status and pain levels do not differ between the treatment groups.

## 2. Methods

### 2.1. Ethics Statement

The local ethics committee approved the study. Written informed consent was obtained from the patients. The study conforms to the Declaration of Helsinki and was registered in the German Clinical Trials Register (DRKS00003591).

### 2.2. Patients

**Figure 1:**
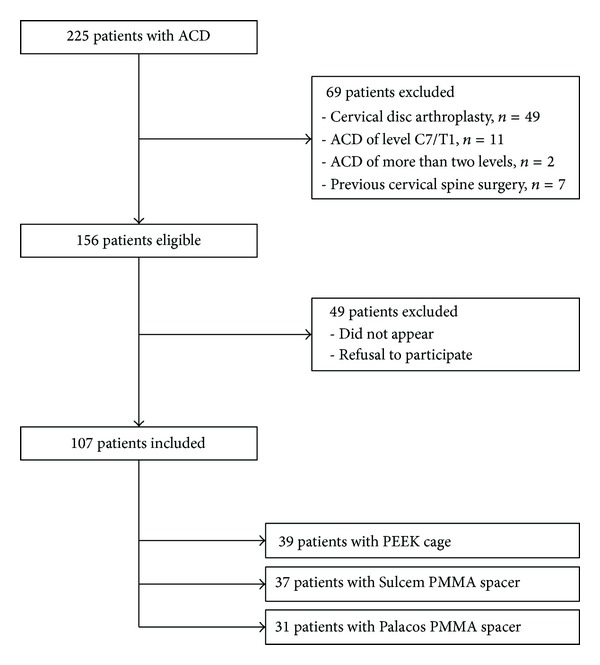
Patient flow diagram. Out of 225 patients with ACD between January 2005 and February 2009, 107 patients were included in one PEEK cage group and two PMMA groups. ACD: anterior cervical discectomy, PEEK: polyetheretherketone, and PMMA: polymethylmethacrylate.

We retrospectively identified 225 patients in our database of a single center who underwent ACD without anterior plating for cervical degenerative disc disease with or without posterior osteophytes between January 2005 and February 2009. Of these, 69 patients were excluded due to implantation of disc prosthesis, previous cervical spine surgery, and ACD of level C7/T1 or more than two cervical levels. 107 of the remaining 156 patients could be contacted to participate in the study (Figure 1).

### 2.3. Surgical Treatment

A standard right-sided anterior approach was performed for ACD in supine position with complete excision of the intervertebral disc. Posterior osteophytes and herniated disc fragments were resected microsurgically. Moreover, the neural foramens were decompressed on both sides, and the posterior longitudinal ligament was dissected and removed. After careful curettage of subchondral cartilage while preserving intact endplates, either a PEEK cage without additional filling or a PMMA spacer (Sulcem, Zimmer Germany GmbH, Freiburg, Germany; Palacos, Heraeus Medical GmbH, Wehrheim, Germany) was implanted into the intervertebral disc space. The optimal cage size was determined under lateral fluoroscopic guidance for restoring disc height and cervical lordosis. In the PMMA groups, absorbable gelatin sponges were used to protect the nerve roots and dura against thermic injury. Additionally, two small asymmetric holes were drilled in the middle of both endplates to prevent slippage of the hardened PMMA spacer.

### 2.4. Postoperative Care

Postoperative external collar fixation was routinely applied for two weeks. On the first postoperative day, the patients were mobilized under physiotherapeutic guidance.

### 2.5. Radiographic Assessment

Plain radiographs in anterior-posterior and lateral projections were obtained before surgery, after surgery before discharge, and at follow-up (at least 12 months postoperatively) according to the protocol of our department. Additionally at follow-up, a thin-sliced CT scan of the treated and adjacent levels was performed routinely. Two surgeons blinded to the clinical status of the patient assessed the digital radiographs and CT scans using integrated software to measure distances and angles (IMPAX EE R20 VIII, Agfa HealthCare, Mortsel, Belgium). The mean values of the two surgeons' measurements were used for further analysis. In case of conflicting evaluation of the fusion status, the surgeons reassessed the CT scan and came to an agreement.

**Figure 2:**
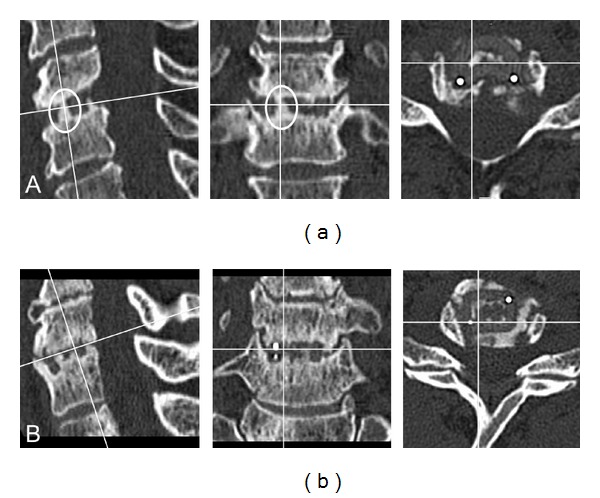
Fusion and nonfusion. Postoperative three-dimensional reconstructed CT scans after ACD with implantation of PEEK cages. (a) A continuous trabecular bone bridge through the cage (encircled) confirmed bony fusion; (b) no bony fusion was assessed due to the lack of continuous trabecular bone bridges.

*Fusion* was determined in three-dimensional reconstructed CT scans and confirmed if continuous trabecular bone bridges through or around the implant were clearly present (Figure 2).

**Figure 3:**
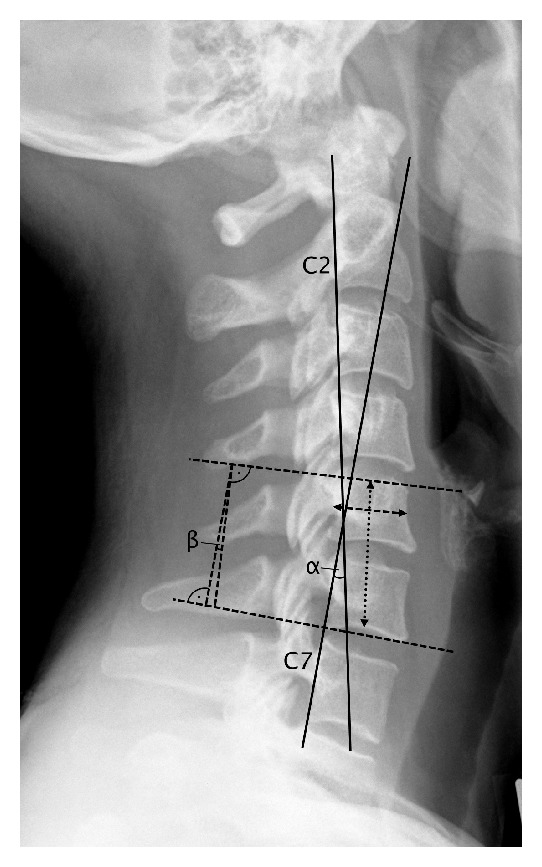
Measurement techniques for subsidence and sagittal alignments. The interbody height ratio of C5/6 is the total segmental height (vertical dotted line with arrowheads) divided by the anterior-posterior diameter of C5 (horizontal dashed line with arrowheads). *α* is the angle indicating the cervical sagittal alignment (CSA); *β* is the angle indicating the segmental sagittal alignment (SSA).

*Subsidence* and* sagittal alignments* were measured in lateral radiographs. For evaluating subsidence, we measured the total segmental height, which includes the central heights of the two vertebras and the disc space of the treated level (Figure 3). The difference between the total segmental height at follow-up and after surgery before discharge was considered as subsidence provided that the vertebral bodies showed no reduced height, for example, due to vertebral fractures. Subsidence was indicated as positive values. Furthermore we assessed the interbody height ratio [21, 29], which is the total segmental height divided by the anterior-posterior diameter of the upper vertebral body and hereby eliminates magnification variation in radiographs (Figure 3).

For evaluating the* segmental sagittal alignment (SSA)*, we applied the Cobb angle. For evaluating the* cervical sagittal alignment (CSA)*, the angle of the tangent to the C2 and C7 posterior vertebral body margins was measured [30] (Figure 3). Changes in SSA and CSA were calculated as difference of the values between follow-up and after surgery before discharge. Change toward kyphosis was indicated as negative values.

### 2.6. Clinical Assessment

The patients' subjective condition at follow-up was obtained with the Visual Analog Scale (VAS) for pain with a range of 0–10 (0: no pain; 10: worst possible pain), Neck Disability Index (NDI) with a range of 0–100 (0: no functional disability; 100: complete functional disability), and Short Form 36 Health Survey (SF-36) with a range of 0–100 (0: worst scale value; 100: best scale value); physical and mental component summaries have been normalized to a mean of 50 and standard deviation of 10 to assess levels of pain, body function, and quality of life.

To evaluate patients' satisfaction with the postoperative result, the Patient Satisfaction Index was applied at follow-up [31]. The Patient Satisfaction Index is a modified subitem of the North American Spine Society outcome questionnaire. It is scored as follows: (1) “Surgery met my expectations”; (2) “I did not improve as much as I had hoped but I would undergo the same operation for the same results”; (3) “Surgery helped but I would not undergo the same operation for the same results”; and (4) “I am the same or worse as compared to before surgery.”

### 2.7. Statistical Analysis

Results were expressed as mean with standard deviations. Analysis of independent continuous quantitative variables between groups was performed using the two-tailed Student's* t*-test. Statistical comparisons for categorical values between groups were accomplished using the two-tailed Fisher exact test and the *χ* ^2^ test. Pearson's correlation was used for regression analysis to evaluate the relationship between subsidence and change in SSA and between change in SSA and CSA. Prism 6 for Mac (GraphPad Software Inc., La Jolla, USA) and Excel 2011 for Mac (Microsoft Corporation, Redmond, USA) were used as statistical software and for data processing. *P* values <0.05 were considered to be statistically significant.

## 3. Results

### 3.1. Patients

The study analyzed 107 patients (50 female, 57 male) who underwent ACD and implantation of a PEEK cage or PMMA spacer (Table 1). The mean age was 55 years (range: 29–82 years). Radicular symptoms were present in 87 patients (81.3%) and myelopathic symptoms were present in 25 patients (23.4%). 73 patients were operated on in one level and 34 patients in two levels leading to a total of 141 operated levels. Soft disc herniation was observed in 44 operated levels (31.2%), spinal canal stenosis in 67 levels (47.5%), and a combination of both in 30 levels (21.3%).

A cervical PEEK cage was implanted in 39 patients (51 levels), a Sulcem PMMA spacer in 37 patients (49 levels), and a Palacos PMMA spacer in 31 patients (41 levels). Different types of PEEK cages were used depending on surgeons' preference (14× C-MAXX, 12× Blackstone, 10× Arca Medica, 10× Acromed Depuy, 2× Solis Stryker, 2× Medicrea Impix-C, and 1× Shell-Cage). A majority of the patients were treated at C5/6. The follow-up times of the groups were significantly different (PEEK cage group: 16 ± 3 months; Sulcem PMMA group: 46 ± 8 months; Palacos PMMA group: 27 ± 7 months) with a mean of 29 ± 14 months (range: 12–57 months).

### 3.2. Radiographic Evaluation

#### 3.2.1. Fusion

Bony fusion was confirmed in CT scans (Figure 2) in 64.6% of treated levels in the PEEK cage group, 57.1% of treated levels in the Sulcem PMMA group, and 46.3% of treated levels in the Palacos PMMA group. There was no statistically significant difference of the fusion status between the treatment groups (Table 2).

#### 3.2.2. Subsidence

Lateral radiographs showed a mean subsidence from directly postoperative to follow-up of 2.3 mm in the PEEK cage group, 2.6 mm in the Sulcem PMMA group, and 2.3 mm in the Palacos PMMA group without being statistically significant between the groups (Table 2). Comparing the clinical outcome between the subgroups with a subsidence ≥3 mm versus <3 mm within each group, there were no significant differences except for the PEEK cage group; herein, the pain scores (VAS: 1.7 ± 1.2 versus 3.9 ± 1.8; bodily pain (SF-36): 57 ± 21 versus 37 ± 13) and physical function (SF-36; 78 ± 20 versus 58 ± 25) showed better outcome in the subgroup with a subsidence ≥3 mm.

The interbody height ratio [21] at follow-up compared to directly postoperative was 0.91 ± 0.08 (PEEK cage), 0.87 ± 0.08 (Sulcem), and 0.90 ± 0.08 (Palacos).

#### 3.2.3. Change in SSA and CSA

Change in SSA toward kyphosis of the treated level from directly postoperative to follow-up was the highest in the PEEK cage group (−4.1°), followed by the Palacos PMMA group (−2.4°) and the Sulcem PMMA group (−1.0°), yielding a statistically significant higher loss of segmental lordosis in the PEEK cage group compared to the Palacos PMMA group (*P* = 0.003) (Table 2).

Change in CSA toward kyphosis between C2 and C7 was −3.1° in the PEEK cage group, −5.2° in the Sulcem PMMA group, and −1.3° in the Palacos PMMA group without showing statistically significant differences.

### 3.3. Clinical Outcome

At follow-up, the mean VAS pain score was 3.1 in the PEEK cage group, 3.6 in the Sulcem PMMA group, and 2.7 in the Palacos PMMA group without significant differences between the groups (Table 3).

The mean NDI score was 26.5 ± 15.8 (range: 0–66) in the PEEK cage group, 34.7 ± 18.9 (range: 2–84) in the Sulcem PMMA group, and 24.9 ± 17.3 (range: 0–62) in the Palacos PMMA group with a statistically significant better NDI in the Palacos PMMA group compared to the Sulcem PMMA group (*P* = 0.034). Furthermore, the PEEK cage group showed a nearly statistically significant better NDI compared to the Sulcem PMMA group (*P* = 0.051) (Table 3).

Statistical analysis of the SF-36 revealed statistically significant worse physical function and physical component summary of the Sulcem PMMA group (49.7 and 33.4) compared to the PEEK cage (67.5 and 39.1) and Palacos PMMA group (63.2 and 39.4) (*P* < 0.05). The remaining scales of the SF-36 showed no statistically significant differences (Table 3).

The Patient Satisfaction Index as evaluation of patients' satisfaction with the postoperative result led to the following answers: “1” (46% PEEK cage, 32% Sulcem, and 48% Palacos); “2” (35% PEEK cage, 35% Sulcem, and 23% Palacos); “3” (5% PEEK cage, 9% Sulcem, and 23% Palacos); and “4” (14% PEEK cage, 24% Sulcem, and 6% Palacos). *χ* ^2^ test revealed no statistical difference of the Patient Satisfaction Index between the treatment groups (*P* = 0.118).

### 3.4. Comparisons

#### 3.4.1. Fused Levels and Change in SSA

Within the PEEK cage group, fused levels showed statistically significant higher changes in SSA toward kyphosis compared to nonfused levels (−5.2° versus −2.1°; *P* = 0.030) (Table 4). The Sulcem and Palacos PMMA groups revealed no statistically significant differences.

#### 3.4.2. Fused Patients and Outcome

Patients were classified as “fused” if all treated levels showed fusion and as “nonfused” if at least one level displayed no fusion. In this respect, 62.2% of the patients in the PEEK cage group were classified as fused, 51.3% in the Sulcem PMMA group, and 41.9% in the Palacos PMMA group (Table 2).

Analysis between fused and nonfused patients in respect to clinical outcome revealed that, in the Sulcem PMMA group, fused patients showed a statistically significant better physical component summary of the SF-36 than nonfused patients (*P* = 0.024) (Table 4). Interestingly, the fused subgroups in the PEEK cage and the Palacos PMMA groups did not even present a numerical improvement of the physical component summary or physical function compared to their nonfused subgroups. The remaining subitems of the SF-36 showed no statistically significant differences between fused and nonfused subgroups in respect to clinical outcome (data not shown). Fused patients in all substitute groups showed lower (better) VAS and NDI scores than the nonfused subgroups though without being statistically significant (Table 4).

#### 3.4.3. Subsidence and Fusion: Subsidence and Change in SSA

There was no statistical difference with regard to fusion status between levels with subsidence of at least 3 mm compared to less than 3 mm (Table 5). Overall, fused levels displayed a higher mean subsidence (3.0 mm) than nonfused levels (2.1 mm) without reaching statistical significance (*P* = 0.055) (Table 4).

The mean change in SSA did not differ significantly between the subsidence categories with the threshold of 3 mm (Table 5).

### 3.5. Correlation Analyses

#### 3.5.1. Subsidence and Change in SSA

To compare the effect of subsidence on the change in SSA, regression analyses were conducted. Subsidence was not a significant predictor of a change in SSA in any group (PEEK cage: *R* ^2^ = 0.003 and *P* = 0.741; Sulcem: *R* ^2^ = 0.014 and, *P* = 0.449; Palacos: *R* ^2^ = 0.012 and *P* = 0.535).

#### 3.5.2. SSA and CSA

**Figure 4:**
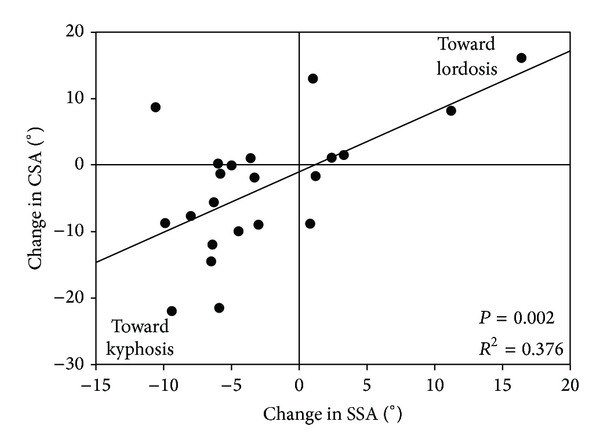
Correlation between SSA and CSA after ACD with implantation of Sulcem PMMA spacers. The diagram shows the positive correlation between SSA and CSA in the Sulcem PMMA group indicating that a change in SSA was a significant predictor of a change in CSA. The mean change in SSA was −2.4° and in CSA was −5.2°. ACD: anterior cervical discectomy, CSA: cervical sagittal alignment, PMMA: polymethylmethacrylate, and SSA: segmental sagittal alignment.

Correlation analysis between change in SSA and CSA was performed on patients solely operated on in one level to ensure homogeneous patient groups. Change in SSA was a significant predictor of a change in CSA in the Sulcem (*R* ^2^ = 0.376 and *P* = 0.002; Figure 4) and Palacos PMMA groups (*R* ^2^ = 0.264 and *P* = 0.042) but not in the PEEK cage group (*R* ^2^ = 0.064 and *P* = 0.223).

### 3.6. Reoperations

In the PEEK cage group, one patient experienced recurrent radicular pain and was reoperated 3 months after ACD due to anterior cage dislocation at level C5/6 and a new soft prolapse at level C6/7.

In the Sulcem PMMA group, two patients underwent revision surgery. One patient was relieved of newly developed radicular pain by unilateral posterior foraminotomy at level C7/T1 two years after ACD at level C6/7, which was now fused. Another patient developed new radicular pain with paresthesia one year after ACD at levels C4/5 (nonfused) and C5/6 (fused) due to neuroforaminal stenosis; unilateral posterior foraminotomy at level C5/6 led to pain elimination.

In the Palacos PMMA group, one patient had to be surgically revised 18 months after ACD at level C3/4 (nonfused) due to retrospondylosis of C4 with spinal canal stenosis and kyphotic malalignment. Cervical alignment was restored by decompressive corpectomy of C4 and fusion with tricortical iliac crest autograft and anterior plating. 

## 4. Discussion

### 4.1. Fusion

At the first sight, the present study displays comparatively high nonfusion rates after ACD in all treatment groups. The impact of using thin-sliced CT scans instead of lateral radiographs for assessment of bony fusion will be discussed below. However, higher fusion rates were not reflected in better clinical outcome except for Sulcem PMMA spacers with a significantly worse outcome in nonfused patients.

ACD with implantation of substitutes is a widely accepted surgical technique in cervical degenerative disc disease [1]. Intervertebral cages and PMMA spacers demonstrated good clinical outcome with differing fusion rates [1–3, 5, 12, 15–18]. Titanium cages filled with additional different materials revealed fusion rates of 47–97% [5, 16, 17, 32, 33]. Empty titanium cages showed fusion rates of 87% [2, 13]. Two comparative studies found higher fusion rates in filled PEEK cages than in filled titanium cages [14, 16]. In contrast, Cabraja et al. recently stated bone formation in 80% using empty titanium cages and 62% using empty PEEK cages [18]. But it has to be considered that a reliable assessment of trabecular bone formation and radiographic fusion signs is prevented in radiopaque titanium cages [23]. Besides the radiolucency of PEEK [15, 17, 22], a further advantage over titanium is supposed to be the elastic modulus of PEEK which is similar to that of cortical bone and is assumed to prevent cage subsidence [18, 20, 23, 29]. Since cervical vertebral endplates are thin layers of subchondral bone [34] and not cortical bone, the impact of this characteristic remains unclear.

PEEK cages filled with additional different materials showed fusion rates of 89–100% [14, 16, 20, 22–26, 35, 36]. Only few studies examined empty PEEK cages and reported bony fusion rates of 62% [18], 72% [19], and 76% (30% with “obvious fusion” and 46% with “probable fusion,” only 6-month follow-up) [21], which are comparable with the present study.

PMMA spacers showed fusion rates of 0–66% [2–6, 37, 38], in an old publication by Böker et al. [7] of even 89%, though using radiographs for evaluation of bony fusion. Therefore, the fusion rates of the PMMA groups in the present study are in line with the published data.

The technique for assessment of bony fusion varies between the studies. Most authors use lateral radiographs [2, 3, 13, 14, 16–23, 25, 26, 33, 36], though CT imaging is clearly superior [22, 27] and was used in only few trials [5, 35]. We used thin-sliced CT scans with multiplanar reconstruction for the highest accuracy in assessing bony fusion. This might imply overestimation of bony fusion in previous studies due to inaccuracy in the measurement technique [27]. Therefore, comparisons of studies with different techniques have to be drawn very carefully. Nevertheless, our findings with higher fusion rates in PEEK cages (65%) than in Sulcem (57%) and Palacos (46%) PMMA spacers are in line with other reports showing higher fusion in titanium cages than in PMMA spacers (87% versus 66%, 2-year follow-up [2]; 97% versus 0%, 1-year follow-up [5]). In consequence of fusion rates of 65% and less, we prefer to use the term* ACD with implantation of a substitute* instead of* ACD and fusion (ACDF)*.

The higher rate of nonfusion in PMMA spacers could be attributed to a hindered ossification that can only develop around the PMMA spacer and not through the PMMA itself in contrast to a hollow cage [3, 6]. Moreover, missing attachment of PMMA to the bone with formation of a fibrous cement-bone interface was stated as a reason for nonfusion [6].

The clinical outcome, however, was generally not influenced by fusion status (Table 4), which was also observed by other authors [3, 18, 19, 21]. Only the Sulcem PMMA group showed a significantly better physical outcome in fused than in nonfused patients (Table 4). Although the Sulcem PMMA group revealed worse functional outcome than the PEEK cage and Palacos PMMA group, no clear beneficial substitute could be specified. Other studies found no difference in the clinical outcome between different substrates like titanium cages and PMMA [1, 2, 5, 6, 8, 37] or titanium and PEEK cages [18].

### 4.2. Subsidence

The loss of about 10% of the interbody height ratio in our patients over the course of time is consistent with results of other studies (up to 7.2% [21] and 9.2% [39] in PEEK cages). In addition, the overall mean subsidence (Table 4) is comparable to the results of Pechlivanis et al. [19] who reported a mean PEEK cage subsidence of 2.9 mm in fused and of 1.5 mm in nonfused patients. They found that the fusion group revealed a significant higher subsidence in PEEK cages [19]. Our data tended to correspond to their findings only after pooling all groups, though without reaching statistical significance (Tables 4 and 5). Why higher subsidence might promote fusion is unclear, but a broader contact of the substitute to cancellous bone and therefore promoting bone growth inducing factors were postulated as one reason [19].

In a biomechanical in vitro study, bone cement was found to exhibit a significantly lower subsidence than titanium or carbon fiber cages [40]. Besides, the elastic module of PEEK was hypothesized to be too flexible and could therefore lead to endplate failure with subsidence [19]. Our data with similar subsidence in the PEEK cage and PMMA groups conflict this hypothesis.

The incidence of subsidence (threshold in our study of 3 mm) was 36% in each group (Table 5). Other studies reported differing incidences of subsidence of 56% [41] (titanium cages, threshold of 3 mm); 44% [12] and 20% [18] (titanium cages, threshold of 2 mm); 15% [27] and 13% [19] (PEEK cages, threshold of 3 mm); and 29% [21], 26% [27], and 14% [18] (PEEK cages, threshold of 2 mm).

One patient in the Sulcem PMMA group needed further surgery with decompression of the neuroforamen on one side, which can be attributed to severe subsidence after ACD. No further associations between higher subsidence and worse clinical outcome were found. In the PEEK cage group, patients with a subsidence of at least 3 mm even showed a better outcome, which is an unexpected result and inconsistent with the general understanding. As usual, in an academic center, several surgeons with different surgical skills and techniques operated on this patient cohort. This fact could have contributed to these unexpected findings. Other authors did not find significantly differing clinical outcome measures between subsidence and nonsubsidence groups [42].

### 4.3. Sagittal Alignment

The highest loss of lordosis (negative change in SSA) was found in the PEEK cage group and was significantly higher than in the Palacos PMMA group (Table 2). PEEK cages also had a significantly higher loss of lordosis in fused levels than in nonfused levels (Table 4) supposing that a higher loss of lordosis might promote bony fusion while bringing the anterior aspects of the endplates and their fracture fragments closer together. Moreover, in the PEEK cage group, the subgroup with a subsidence of at least 3 mm showed a higher, though not significant, loss of lordosis than the subgroup with a subsidence less than 3 mm (−5.5° versus −3.4°; Table 5). In the PMMA groups, however, these observations were not reflected.

Like in the Sulcem and Palacos PMMA groups of the present study, also prior studies showed a significant positive correlation of a change in SSA and CSA after ACD with filled PEEK cages [22].

### 4.4. Limitations of the Study

The retrospective design is an obvious methodological weakness of the study. Since patients were retrospectively included, no power analysis was performed. We intended to reduce a possible selection bias with precise patient selection criteria. The heterogeneity of patients with radicular and myelopathic symptoms can lead to bias in clinical outcome. In line with the retrospective design, differing follow-up times of the treatment groups come, which themselves can contribute to bias complication rates or radiographic measurements.

## 5. Conclusions

There are different fusion rates after ACD with implantation of PEEK cages and PMMA spacers. The results of the current study might indicate, in agreement with other reports, that fusion status and subsidence do not correlate with clinical outcome. No clear advantageous disc substitute could be specified; however, the PEEK cage and Palacos PMMA groups appeared to present better function in comparison to the Sulcem PMMA group.

## Figures and Tables

**Table 1:** Selected demographic and clinical data.

	PEEK cage	PMMA Sulcem	PMMA Palacos	Overall	*P* (cage versus Sulcem)	*P* (cage versus Palacos)	*P* (Sulcem versus Palacos)
Number of patients	39	37	31	107			
Mean age (yr)^#^	53	57	57	55	0.096	0.130	0.759
Male : female ratio^∧^	20 : 19	23 : 14	14 : 17	57 : 50	0.364	0.638	0.222
One level : two level ratio^∧^	27 : 12	25 : 12	21 : 10	73 : 34	1.000	1.000	1.000
Radiculopathy^∧^	31/39	33/37	23/31	87/107	0.348	0.775	0.124
Myelopathy^∧^	10/39	5/37	10/31	25/107	0.252	0.601	0.082
C3/4^†^	2	5	5	12	0.265	0.505	0.612
C4/5	8	3	5	16			
C5/6	22	25	16	63			
C6/7	19	16	15	50			

The table shows demographics and clinical data with distribution of surgery levels.

^#^Student's *t*-test (two-tailed).

^∧^Fisher exact test (two-tailed).

^†^ *χ* ^2^ test.

**Table 2:** Bony fusion, subsidence, and sagittal alignment.

	PEEK cage	PMMA Sulcem	PMMA Palacos	Overall	*P* (cage versus Sulcem)	*P* (cage versus Palacos)	*P* (Sulcem versus Palacos)
Fused levels^∧^	31/48 (64.6%)	28/49 (57.1%)	19/41 (46.3%)	78/138 (56.5%)	0.534	0.092	0.397
Fused patients^∧^	23/37 (62.2%)	19/37 (51.3%)	13/31 (41.9%)	55/105 (52.4%)	0.482	0.143	0.474
Subsidence (mm)^#^	2.3 ± 2.8	2.6 ± 3.1	2.3 ± 2.9	2.4 ± 3.0	0.647	0.948	0.721
Change in SSA^#^	−4.1° ± 4.3°	−2.4° ± 6.2°	−1.0° ± 4.6°	−2.7°	0.129	0.003*	0.260
Change in CSA^#^	−3.1° ± 10.1°	−5.2° ± 10.7°	−1.3° ± 10.0°	−3.6°	0.420	0.500	0.168

The table shows ratios of fused levels and fused patients as well as means with standard deviations of subsidence and change in segmental and cervical alignment.

SSA: segmental sagittal alignment.

CSA: cervical sagittal alignment.

^#^Student's *t*-test (two-tailed).

^∧^Fisher exact test (two-tailed).

*P < 0.05.

**Table 3:** Clinical outcome.

	PEEK cage	PMMA Sulcem	PMMA Palacos	Overall	*P* (cage versus Sulcem)	*P* (cage versus Palacos)	*P* (Sulcem versus Palacos)
VAS^#^	3.1 ± 2.0	3.6 ± 2.5	2.7 ± 2.7	3.1 ± 2.4	0.342	0.565	0.193
NDI^#^	26.5 ± 15.8	34.7 ± 18.9	24.9 ± 17.3	28.7 ± 17.7	0.051	0.699	0.034*
SF-36							
Physical function^#^	67.5 ± 24.3	49.7 ± 28.8	63.2 ± 23.8	60.3 ± 26.6	0.007*	0.459	0.044*
Bodily pain^#^	46.4 ± 20.1	42.4 ± 24.0	49.9 ± 30.3	46.2 ± 24.7	0.444	0.588	0.277
General health^#^	53.9 ± 21.4	46.0 ± 20.5	51.5 ± 22.6	50.6 ± 21.5	0.116	0.662	0.309
Vitality^#^	47.2 ± 19.6	42.7 ± 20.8	44.3 ± 20.7	44.8 ± 20.2	0.360	0.557	0.769
Mental health^#^	57.4 ± 21.5	61.8 ± 22.4	65.5 ± 19.8	61.3 ± 21.4	0.403	0.110	0.475
Physical component summary^#^	39.1 ± 9.9	33.4 ± 11.8	39.4 ± 10.9	37.3 ± 11.1	0.036*	0.917	0.044*
Mental component summary^#^	43.3 ± 12.8	44.8 ± 14.5	46.2 ± 11.1	44.6 ± 12.8	0.661	0.335	0.670

The table shows means with standard deviations from self-reported questionnaires.

VAS: Visual Analog Scale.

NDI: Neck Disability Index.

SF-36: Short Form 36 Health Survey.

^#^Student's *t*-test (two-tailed).

*P < 0.05.

**Table 4:** Comparison of sagittal alignment, subsidence, pain level, and functional outcome according to fusion status.

	PEEK cage	PMMA Sulcem	PMMA Palacos	Overall
Change in SSA				
Fused *levels* Nonfused *levels* ^#^ *P*	−5.2° ± 4.4° −2.1° ± 3.9° 0.030*	−2.2° ± 7.4° −3.0° ± 3.8° 0.662	−1.4° ± 3.4° −0.5° ± 6.0° 0.605	−3.3° ± 5.7° −1.8° ± 4.6° 0.133
Subsidence (mm)				
Fused *levels* Nonfused *levels* ^#^ *P*	2.7 ± 2.7 1.7 ± 1.7 0.152	3.2 ± 3.2 2.2 ± 1.8 0.204	3.1 ± 2.1 2.3 ± 2.6 0.336	3.0 ± 2.8 2.1 ± 2.1 0.055
VAS				
Fused *patients* Nonfused *patients* ^#^ *P*	2.9 3.1 0.667	3.4 3.8 0.626	2.0 3.3 0.168	2.8 3.4 0.216
NDI				
Fused *patients* Nonfused *patients* ^#^ *P*	24.1 28.0 0.439	32.7 37.0 0.512	19.9 28.6 0.140	26.0 31.2 0.136
SF-36 Physical function				
Fused *patients* Nonfused *patients* ^#^ *P*	66.4 72.1 0.443	56.4 42.2 0.160	63.0 63.3 0.974	62.2 58.9 0.538
SF-36 Physical component summary				
Fused *patients* Nonfused *patients* ^#^ *P*	39.6 39.7 0.971	37.7 28.5 0.024*	37.8 40.7 0.489	38.5 36.3 0.349

The table shows means (with standard deviations) of change in segmental sagittal alignment, subsidence, pain level, and functional outcome according to the fusion status.

SSA: segmental sagittal alignment.

VAS: Visual Analog Scale.

NDI: Neck Disability Index.

SF-36: Short Form 36 Health Survey.

^#^Student's *t*-test (two-tailed).

*P < 0.05.

**Table 5:** Comparison of fusion and sagittal alignment according to subsidence.

	PEEK cage (*n* = 45)			PMMA Sulcem (*n* = 42)			PMMA Palacos (*n* = 33)			Overall (*n* = 120)		
	*n* (levels)	Fused levels	Change in SSA^†^	*n* (levels)	Fused levels	Change in SSA^†^	*n* (levels)	Fused levels	Change in SSA^†^	*n* (levels)	Fused levels	Change in SSA^†^
Subsidence ≥ 3 mm	16	12/15	−5.5° (r, −15.1–3.5)	15	11/15	−2.0° (r, −15.6–16.4)	12	7/12	0.3° (r, −5.7–15.9)	43	30/42	−2.6° (r, −15.6–16.4)
Subsidence < 3 mm	29	17/28	−3.4° (r, −16.1–2.9)	27	15/27	−2.6° (r, −10.6–11.2)	21	8/21	−1.7° (r, −6.3–7.9)	77	40/76	−2.6° (r, −16.1–11.2)
*P*		0.308^∧^	0.124^#^		0.330^∧^	0.800^#^		0.300^∧^	0.297^#^		0.053^∧^	1.00^#^

The table shows ratios of fused levels and change in segmental sagittal alignment according to the degree of subsidence with a threshold of 3 mm.

^#^Student's *t*-test (two-tailed).

^∧^Fisher exact test (two-tailed).

^†^Negative toward kyphosis; r: range.

## Abbreviations

ACD: Anterior cervical discectomy
CSA: Cervical sagittal alignment
NDI: Neck Disability Index
PEEK: Polyetheretherketone
PMMA: Polymethylmethacrylate
SF-36: Short Form 36 Health Survey
SSA: Segmental sagittal alignment
VAS: Visual Analog Scale.
